# Determinants and prediction of *Chlamydia trachomatis* re-testing and re-infection within 1 year among heterosexuals with chlamydia attending a sexual health clinic

**DOI:** 10.3389/fpubh.2022.1031372

**Published:** 2023-01-13

**Authors:** Xianglong Xu, Eric P. F. Chow, Christopher K. Fairley, Marcus Chen, Ivette Aguirre, Jane Goller, Jane Hocking, Natalie Carvalho, Lei Zhang, Jason J. Ong

**Affiliations:** ^1^Department of Epidemiology and Health Statistics, School of Public Health, Shanghai University of Traditional Chinese Medicine, Shanghai, China; ^2^Melbourne Sexual Health Centre, The Alfred, Melbourne, VIC, Australia; ^3^Central Clinical School, Monash University, Melbourne, VIC, Australia; ^4^Centre for Epidemiology and Biostatistics, Melbourne School of Population and Global Health, The University of Melbourne, Melbourne, VIC, Australia; ^5^Centre for Health Policy, Melbourne School of Population and Global Health, The University of Melbourne, Melbourne, VIC, Australia; ^6^China Australia Joint Research Center for Infectious Diseases, School of Public Health, Xi'an Jiaotong University Health Science Centre, Xi'an, Shaanxi, China; ^7^Faculty of Infectious and Tropical Diseases, London School of Hygiene and Tropical Medicine, London, United Kingdom

**Keywords:** *Chlamydia trachomatis*, re-testing, re-infection, heterosexual, predictive model, machine learning, variable importance, risk factors

## Abstract

**Background:**

*Chlamydia trachomatis* (chlamydia) is one of the most common sexually transmitted infections (STI) globally, and re-infections are common. Current Australian guidelines recommend re-testing for chlamydia 3 months after treatment to identify possible re-infection. Patient-delivered partner therapy (PDPT) has been proposed to control chlamydia re-infection among heterosexuals. We aimed to identify determinants and the prediction of chlamydia re-testing and re-infection within 1 year among heterosexuals with chlamydia to identify potential PDPT candidates.

**Methods:**

Our baseline data included 5,806 heterosexuals with chlamydia aged ≥18 years and 2,070 re-tested for chlamydia within 1 year of their chlamydia diagnosis at the Melbourne Sexual Health Center from January 2, 2015, to May 15, 2020. We used routinely collected electronic health record (EHR) variables and machine-learning models to predict chlamydia re-testing and re-infection events. We also used logistic regression to investigate factors associated with chlamydia re-testing and re-infection.

**Results:**

About 2,070 (36%) of 5,806 heterosexuals with chlamydia were re-tested for chlamydia within 1 year. Among those retested, 307 (15%) were re-infected. Multivariable logistic regression analysis showed that older age (≥35 years old), female, living with HIV, being a current sex worker, patient-delivered partner therapy users, and higher numbers of sex partners were associated with an increased chlamydia re-testing within 1 year. Multivariable logistic regression analysis also showed that younger age (18–24 years), male gender, and living with HIV were associated with an increased chlamydia re-infection within 1 year. The XGBoost model was the best model for predicting chlamydia re-testing and re-infection within 1 year among heterosexuals with chlamydia; however, machine learning approaches and these self-reported answers from clients did not provide a good predictive value (AUC < 60.0%).

**Conclusion:**

The low rate of chlamydia re-testing and high rate of chlamydia re-infection among heterosexuals with chlamydia highlights the need for further interventions. Better targeting of individuals more likely to be re-infected is needed to optimize the provision of PDPT and encourage the test of re-infection at 3 months.

## Background

*Chlamydia trachomatis* (chlamydia) is the most common bacterial sexually transmitted infection (STI) globally (1, 2). An estimated 131 million incident cases of chlamydia infections are acquired globally among people aged 15–49 years annually (3). After treatment, chlamydia re-infections are common, occurring in about 10–20% of patients within 12 months (4–8). Identifying and treating chlamydia re-infection promptly among women is vital because re-infection is associated with a higher risk of pelvic inflammatory disease (9). A study in Australia reported that the chlamydia re-infection rate was 22.3 (95%CI: 13.2–37.6) per 100 person-years among women aged 16–25 years (8). Australian guidelines recommend chlamydia re-testing at 3 months after treatment to identify possible re-infection (10, 11). Chlamydia re-testing was relatively low (40%) within 1.5–12 months (12). Therefore, developing innovative measures that increase repeat testing for chlamydia following chlamydia infection is important.

Treatment of partners is necessary for heterosexuals with chlamydia to prevent re-infection and interrupt the chain of chlamydia transmission (13). Patient Delivery Partner Therapy (PDPT) has been proposed as a possible strategy for reducing re-infection and preventing the sequelae of STIs (14). PDPT involves providing antibiotic treatment to the sexual partners of patients diagnosed with a treatable sexually transmitted infection by giving the prescription or medication to the patient's sexual partner(s) *via* the diagnosed patient (15). The purpose of PDPT is to enable sexual partners who may be reluctant to attend a health provider to be treated earlier, thus reducing the risk of re-infecting the diagnosed patient. Two observational studies found that PDPT may lower women's re-infection risk (16, 17). In Australia, PDPT is permissible for heterosexual men and women diagnosed with chlamydia (18). Identifying heterosexuals with chlamydia at high risk of re-infection who could benefit from PDPT is important for implementing PDPT.

Predictive models can assist clinical decision-making (19). Developing a predictive model for predicting chlamydia re-testing and re-infection within 1 year among heterosexuals could be used to identify potential PDPT candidates and prioritize chlamydia re-infection screening and sexual health service planning. Machine learning approaches have some advantages in prediction, including not requiring statistical inferences and assumptions (20), improving accuracy by exploiting complex interactions between risk factors (21), handling a mass of predictors and combining them in a non-linear and highly interactive way (22). Despite these advantages, to our knowledge, only one study used machine learning to predict STI re-infection. Our study used machine learning algorithms to predict the risk of acquiring ≥1 or ≥2 additional STIs (combinations of gonorrhea, chlamydia, and syphilis) diagnoses within 1 or 2 years after the initial diagnosis among patients (not limited to heterosexuals) in Massachusetts, USA (23). No studies have used machine learning to predict chlamydia re-testing or re-infection among heterosexuals. Our research team has also used machine learning approaches to predict the uptake of HIV/STI testing among men who have sex with men receiving reminders for testing (24).

We aimed to predict chlamydia re-testing and re-infection within 1 year among heterosexuals with chlamydia using machine learning algorithms. We also used univariable and multivariable logistic regression to identify variables associated with chlamydia re-testing and re-infection.

## Methods

Our analysis used electronic health records (EHRs) data between January 2, 2015, and May 15, 2020, at the Melbourne Sexual Health Center (MSHC). We included individuals who were (1) heterosexuals with chlamydia (including heterosexual males and females) and (2) aged 18 years and above.

### Predictors

Based on the literature review and expert discussion, we selected potential predictors. Potential predictors were selected from data routinely collected through a computer-assisted self-interviewing system used when patients enter the clinic (25) and from other routinely collected data items in the clinical setting. Self-reported predictors at baseline (i.e., at the time of their diagnosis of chlamydia) were included, such as demographics (age at the consultation, gender, country of birth, access to Medicare, ever sex worker, current sex worker), sexual practices (numbers of sex partners in the last 3 months, sex overseas in the last 12 months, condoms used with sex partners in the last 3 months), self-reported past STI (i.e., chlamydia, genital herpes, genital warts, gonorrhea, syphilis), HIV infection, use of PDPT (i.e., the diagnosed client receiving an extra prescription of antibiotics to give to their sexual partner), injecting drug use in the last 3 months, triage reason as asymptomatic screening, triage reason as STI symptoms, and triage reason as contact of any STI (summarized in Table 1). In the machine learning analysis, we did not include PDPT users.

**Table 1:** Factors associated with chlamydia re-testing within 1 year among 5,806 heterosexual individuals with chlamydia.

**Characteristic**	**Event rate**	**Crude OR**	**95% CI**	***p*-value**	Adjusted OR^*^	**95% CI**	***p*-value**
**Gender**							
Female	1,089/2,752 (39.6%)	1 (ref)	1 (ref)		1 (ref)	1 (ref)	
Male	981/3,054 (32.1%)	0.72	0.65, 0.80	**<0.001**	0.74	0.66, 0.84	**<0.001**
**HIV infection**							
HIV-negative	2,042/5,762 (35.4%)	1 (ref)	1 (ref)		1 (ref)	1 (ref)	
Living with HIV	28/44 (63.6%)	3.19	1.74, 6.04	**<0.001**	2.27	1.18, 4.50	**0.016**
**Triage reason as asymptomatic screening**							
No	1,910/5,404 (35.3%)	1 (ref)	1 (ref)		1 (ref)	1 (ref)	
Yes	160/402 (39.8%)	1.21	0.98, 1.49	**0.072**	1.04	0.83, 1.29	0.749
**Triage reason as STI symptoms**							
No	1,139/3,235 (35.2%)	1 (ref)	1 (ref)				
Yes	931/2,571 (36.2%)	1.04	0.94, 1.16	0.428			
**Triage reason as contact of STI**							
No	1,703/4,547 (37.5%)	1 (ref)	1 (ref)		1 (ref)	1 (ref)	
Yes	367/1,259 (29.2%)	0.69	0.60, 0.79	**<0.001**	0.79	0.69, 0.91	**0.001**
**Past chlamydia**							
No	1,663/4,678 (35.5%)	1 (ref)	1 (ref)				
Yes	407/1,128 (36.1%)	1.02	0.89, 1.17	0.738			
**Past genital herpes**							
No	2,011/5,655 (35.6%)	1 (ref)	1 (ref)				
Yes	59/151 (39.1%)	1.16	0.83, 1.61	0.374			
**Past genital warts**							
No	1,991/5,610 (35.5%)	1 (ref)	1 (ref)				
Yes	79/196 (40.3%)	1.23	0.92, 1.64	**0.167**			
**Past gonorrhea**							
No	2,015/5,661 (35.6%)	1 (ref)	1 (ref)				
Yes	55/145 (37.9%)	1.11	0.78, 1.55	0.562			
**Past syphilis**							
No	2,061/5,779 (35.7%)	1 (ref)	1 (ref)				
Yes	9/27 (33.3%)	0.90	0.39, 1.96	0.801			
**Current sex worker**							
No	1,773/5,255 (33.7%)	1 (ref)	1 (ref)		1 (ref)	1 (ref)	
Unknown/missing	123/280 (43.9%)	1.54	1.21, 1.96	**<0.001**	1.35	0.81, 2.28	0.250
Yes	174/271 (64.2%)	3.52	2.74, 4.56	**<0.001**	2.50	1.88, 3.33	**<0.001**
**Sex overseas in the past 12 months**							
No	942/2,517 (37.4%)	1 (ref)	1 (ref)		1 (ref)	1 (ref)	
Unknown/missing	188/450 (41.8%)	1.20	0.98, 1.47	**0.080**	1.06	0.78, 1.43	0.703
Yes	940/2,839 (33.1%)	0.83	0.74, 0.93	**<0.001**	0.90	0.79, 1.01	0.077
**Use of PDPT**							
No	1,947/5,540 (35.1%)	1 (ref)	1 (ref)		1 (ref)	1 (ref)	
Yes	123/266 (46.2%)	1.59	1.24, 2.03	**<0.001**	1.32	1.02, 1.70	**0.033**
**Country of birth**							
Australia	587/1,610 (36.5%)	1 (ref)	1 (ref)		1 (ref)	1 (ref)	
Not Australia	1,414/3,987 (35.5%)	0.96	0.85, 1.08	0.482	0.92	0.80, 1.06	0.263
Unknown/missing	69/209 (33.0%)	0.86	0.63, 1.16	0.329	0.79	0.57, 1.09	0.152
**Access to Medicare**							
No	786/1,995 (39.4%)	1 (ref)	1 (ref)		1 (ref)	1 (ref)	
Unknown/missing	155/780 (19.9%)	0.38	0.31, 0.46	**<0.001**	0.41	0.34, 0.50	**<0.001**
Yes	1,129/3,031 (37.2%)	0.91	0.81, 1.03	**0.125**	0.91	0.79, 1.04	0.182
**Age**							
18–24 years	783/2,331 (33.6%)	1 (ref)	1 (ref)		1 (ref)	1 (ref)	
25–34 years	1,044/2,895 (36.1%)	1.12	0.99, 1.25	**0.063**	1.13	1.00, 1.27	0.052
35 years old and above	243/580 (41.9%)	1.43	1.18, 1.72	**<0.001**	1.27	1.04, 1.55	**0.019**
**Injecting drug use in the past 3 months**							
No	1,926/5,440 (35.4%)	1 (ref)	1 (ref)		1 (ref)	1 (ref)	
Unknown/missing	132/320 (41.2%)	1.28	1.02, 1.61	**0.034**	0.83	0.52, 1.29	0.404
Yes	12/46 (26.1%)	0.64	0.32, 1.21	**0.191**	0.54	0.26, 1.06	0.085
**Numbers of sex partners in the past 3 months**							
1 sex partner	497/1,446 (34.4%)	1 (ref)	1 (ref)		1 (ref)	1 (ref)	
2–5 sex partners	983/2,816 (34.9%)	1.02	0.90, 1.17	0.727	1.13	0.98, 1.30	0.083
5–10 sex partners	265/768 (34.5%)	1.01	0.84, 1.21	0.949	1.20	0.99, 1.46	0.064
More than 10 sex partners	76/196 (38.8%)	1.21	0.89, 1.64	0.225	1.46	1.06, 2.01	**0.020**
Unknown/missing	41/137 (29.9%)	0.82	0.55, 1.19	0.295	0.89	0.57, 1.39	0.626
N/A	443						
**Condoms used with sex partners in the last 3 months**							
Always use condom	168/395 (42.5%)	1 (ref)	1 (ref)		1 (ref)	1 (ref)	
Not Always	1643/4,793 (34.3%)	0.70	0.57, 0.87	**<0.001**	0.80	0.65, 1.00	0.052
Unknown/missing	1643/4,793 (34.3%)	0.56	0.38, 0.81	**<0.003**	0.65	0.42, 1.00	0.052
N/A	208/443 (47.0%)						

OR, Odds Ratio; CI, Confidence Interval; N/A, no sex in the past 3 months; PDPT, patient delivery partner therapy.

* Variables with p < 0.20 in the univariable logistic regression analysis were included in the multivariable logistic regression analysis. The bold values indicate variables with p < 0.20 in the univariable logistic regression analysis.

### Outcome

The primary outcome was chlamydia re-infection, defined as the first new chlamydia diagnosis using nucleic acid amplification testing from any anatomical site, including the oropharynx, urethra/urine, or anorectum, at least 30 days after and within 365 days after a positive chlamydia diagnosis. The selection of chlamydia re-infection from 30 to 365 days after a positive chlamydia diagnosis is consistent with a *Chlamydia trachomatis* re-infection study among female adolescents (26). In this study, chlamydia re-testing was defined as those heterosexuals who had new chlamydia diagnosis results (either positive or negative) at least 30 days after and within 365 days after a positive chlamydia diagnosis.

### Analysis

Statistical analysis was conducted with R 4.0.3. Descriptive statistics were used to summarize the patient characteristics. We also performed risk factor analysis using univariable and multivariable logistic regression analyses to calculate the unadjusted odds ratio (OR) and adjusted OR. Variables with *p* < 0.20 in the univariable logistic regression analysis were included in the multivariable logistic regression analysis to identify independent risk factors. All statistics were performed using a two-sided test, and statistical significance was considered at *p* < 0.05.

Machine learning algorithms were conducted with Python 3.9.7. We developed 10 commonly utilized machine learning models, including Logistic Regression (LR), K-Nearest Neighbors (KNN), Adaptive Boosting Classifier (AdaBoost), SVM with a Radial Basis Function Kernel (SVM), Gaussian Naive Bayes (GaussianNB), Gradient Boosting Machine (GBM), Light Gradient Boosting Decision Machine (LightGBM), Extreme Gradient Boosting (XGBoost), Random Forest (RF), and Multi-Layer Perceptron (MLP). LR, GBM, AdaBoost, GaussianNB, KNN, SVM, RF, and MLP was built using the *scikit-learn* library. LightGBM was built using the *LightGBM* library. XGBoost was built using the *xgboost* library. We performed a 3 × 10 (3 outer folds, 10 inner folds) nested cross-validation to avoid overfitting and improve generalizability (25, 27). We used the Bayesian optimisation method for tuning the hyperparameters. The performance of machine learning models was measured by the area under the receiver operating characteristics curve (AUC) and accuracy on the independent testing dataset. We also calculated the variable importance of chlamydia re-testing and re-infection within 1 year to investigate the effect of different predictors on prediction (24, 25, 28). First, we obtained the results of variable importance analysis from each fold in the outer loop of nested cross-validation. Then based on the value of AUC, we chose the one closest to the mean AUC and reported the results of this variable importance analysis.

### Ethics approval

Ethical approval was granted by the Alfred Hospital Ethics Committee, Australia (project number: 277/20).

## Results

### Characteristics of the study population

Our study data included 5,806 heterosexual patients diagnosed with chlamydia. Among 5,806 patients, 35.7% (2,070/5,806) were re-tested for chlamydia within 1 year. A total of 14.8% (307/2,070) heterosexuals with chlamydia were re-infected with chlamydia within 1 year. Further details of the sociodemographic characteristics and sexual practices are provided in Tables 1, 2.

**Table 2:** Factors associated with chlamydia re-infection within 1 year among 2,070 re-tested heterosexual individuals.

**Characteristic**	**Event rate**	**Crude OR**	**95% CI**	***p*-value**	Adjusted OR^*^	**95% CI**	***p*-value**
**Gender**							
Female	132/1,089 (12.1%)	1 (ref)	1 (ref)		1 (ref)	1 (ref)	
Male	175/981 (17.8%)	1.57	1.23, 2.01	**<0.001**	1.55	1.16, 2.08	**0.003**
**HIV infection**							
HIV-negative	294/2,042 (14.4%)	1 (ref)	1 (ref)		1 (ref)	1 (ref)	
Living with HIV	13/28 (46.4%)	5.15	2.39, 11.0	**<0.001**	4.02	1.64, 9.87	**0.002**
**Triage reason as asymptomatic screening**							
No	289/1,910 (15.1%)	1 (ref)	1 (ref)				
Yes	18/160 (11.2%)	0.71	0.42, 1.15	**0.187**			
**Triage reason as STI symptoms**							
No	162/1,139 (14.2%)	1 (ref)	1 (ref)				
Yes	145/931 (15.6%)	1.11	0.87, 1.42	0.390			
**Triage reason as contact of STI**							
No	251/1,703 (14.7%)	1 (ref)	1 (ref)				
Yes	56/367 (15.3%)	1.04	0.75, 1.42	0.799			
**Past chlamydia**							
No	247/1,663 (14.9%)	1 (ref)	1 (ref)				
Yes	60/407 (14.7%)	0.99	0.73, 1.34	0.955			
**Past genital herpes**							
No	303/2,011 (15.1%)	1 (ref)	1 (ref)		1 (ref)	1 (ref)	
Yes	4/59 (6.78%)	0.41	0.12, 1.01	**0.087**	0.40	0.12, 1.00	0.085
**Past genital Warts**							
No	293/1,991 (14.7%)	1 (ref)	1 (ref)				
Yes	14/79 (17.7%)	1.25	0.66, 2.19	0.462			
**Past gonorrhea**							
No	295/2,015 (14.6%)	1 (ref)	1 (ref)		1 (ref)	1 (ref)	
Yes	12/55 (21.8%)	1.63	0.81, 3.03	**0.143**	1.65	0.80, 3.18	0.150
**Past syphilis**							
No	306/2,061 (14.8%)	1 (ref)	1 (ref)				
Yes	1/9 (11.1%)	0.72	0.04, 3.93	0.754			
**Current sex worker**							
No	249/1,773 (14.0%)	1 (ref)	1 (ref)		1 (ref)	1 (ref)	
Unknown/missing	30/123 (24.4%)	1.97	1.26, 3.01	**0.002**	0.55	0.17, 1.81	0.334
Yes	28/174 (16.1%)	1.17	0.75, 1.77	0.461	1.22	0.71, 2.04	0.456
**Sex overseas in the past 12 months**							
No	137/942 (14.5%)	1 (ref)	1 (ref)		1 (ref)	1 (ref)	
Unknown/missing	37/188 (19.7%)	1.44	0.95, 2.13	**0.076**	0.61	0.28, 1.24	0.199
Yes	133/940 (14.1%)	0.97	0.75, 1.25	0.807	0.91	0.69, 1.20	0.501
**Use of PDPT**							
No	285/1,947 (14.6%)	1 (ref)	1 (ref)				
Yes	22/123 (17.9%)	1.27	0.77, 2.01	0.327			
**Country of birth**							
Australia	97/587 (16.5%)	1 (ref)	1 (ref)		1 (ref)	1 (ref)	
Not Australia	199/1,414 (14.1%)	0.83	0.64, 1.08	**0.160**	0.87	0.63, 1.20	0.387
Unknown/missing	11/69 (15.9%)	0.96	0.46, 1.82	0.902	1.11	0.52, 2.18	0.772
**Access to Medicare**							
No	116/786 (14.8%)	1 (ref)	1 (ref)		1 (ref)	1 (ref)	
Unknown/missing	16/155 (10.3%)	0.66	0.37, 1.13	**0.148**	0.61	0.34, 1.05	0.088
Yes	175/1,129 (15.5%)	1.06	0.82, 1.37	0.656	0.91	0.66, 1.23	0.533
**Age**							
18**–**24 years	125/783 (16.0%)	1 (ref)	1 (ref)		1 (ref)	1 (ref)	
25**–**34 years	148/1,044 (14.2%)	0.87	0.67, 1.13	0.289	0.78	0.60, 1.02	0.074
35 years old and above	34/243 (14.0%)	0.86	0.56, 1.28	0.458	0.58	0.37, 0.89	**0.016**
**Injecting drug use in the past 3 months**							
No	272/1,926 (14.1%)	1 (ref)	1 (ref)		1 (ref)	1 (ref)	
Unknown/missing	34/132 (25.8%)	2.11	1.38, 3.15	**<0.001**	2.11	0.73, 5.55	0.144
Yes	1/12 (8.33%)	0.55	0.03, 2.86	0.571	0.30	0.01, 1.95	0.306
**Numbers of sex partners in the past 3 months**							
1 sex partner	64/497 (12.9%)	1 (ref)	1 (ref)		1 (ref)	1 (ref)	
2**–**5 sex partners	129/983 (13.1%)	1.02	0.74, 1.42	0.894	1.02	0.73, 1.42	0.927
5**–**10 sex partners	47/265 (17.7%)	1.46	0.96, 2.19	**0.071**	1.38	0.90, 2.11	0.140
More than 10 sex partners	15/76 (19.7%)	1.66	0.87, 3.04	**0.109**	1.45	0.74, 2.71	0.261
Unknown/missing	3/41 (7.32%)	0.53	0.13, 1.53	0.307	0.51	0.12, 1.51	0.287
N/A	208						
**Condoms used with sex partners in the last 3 months**							
Always use condom	19/168 (11.3%)	1 (ref)	1 (ref)				
Not Always	230/1,643 (14.0%)	1.28	0.80, 2.16	0.336			
Unknown/missing	9/51 (17.6%)	1.68	0.68, 3.90	0.239			
N/A	208						

OR, Odds Ratio; CI, Confidence Interval; N/A, no sex in the past 3 months; PDPT, patient delivery partner therapy.

* Variables with p < 0.20 in the univariable logistic regression analysis were included in the multivariable logistic regression analysis. The bold values indicate variables with p < 0.20 in the univariable logistic regression analysis.

### Factors associated with chlamydia re-testing and chlamydia re-infection within 1 year using univariable and multivariable logistic regression analyses

The potential factors that were included in the multivariable logistic regression analysis of chlamydia re-testing within 1 year among heterosexuals with chlamydia were age, gender, HIV infection, triage reason as asymptomatic screening, triage reason as contact of STI infection, current sex work, use of PDPT, country of birth, access to Medicare, injecting drug use in the past 3 months, had sex with a partner in the past 3 months, numbers of sex partners in the past 3 months, condoms used with sex partners in the last 3 months. Chlamydia re-testing within 1 year among heterosexuals was associated with male gender [adjusted odds ratios (aOR) = 0.74, 95%CI 0.66, 0.84], living with HIV (aOR = 2.27, 95%CI 1.18, 4.50), presentation as contact of STI infection (aOR = 0.79, 95%CI 0.69, 0.91), being a current sex worker (aOR = 2.50, 95%CI 1.88, 3.33), users of patient delivered partner therapy (aOR = 1.32, 95%CI 1.02, 1.70), older age (≥35 years old vs. 18–24 years, aOR = 1.27, 95%CI 1.04, 1.55), unknown/missing on accessing to Medicare (aOR = 0.41, 95%CI 0.34, 0.50), and higher numbers of partners in the last 3 months (>10 vs.1, aOR = 1.46, 95%CI 1.06, 2.01) (Table 1).

The potential factors that were included in the multivariable logistic regression analysis of chlamydia re-infection within 1 year among heterosexuals with chlamydia were age, gender, HIV infection, past genital herpes, past gonorrhea, current sex worker, sex overseas in the past 12 months, country of birth, access to Medicare, injecting drug use in the past 3 months, had sex with a partner in the past 3 months, numbers of sex partners in the past 3 months, and condoms used with sex partners in the past 3 months. Chlamydia re-infection within 1 year among heterosexuals was associated with male gender (aOR = 1.55, 95%CI 1.16, 2.08), living with HIV (aOR = 4.02, 95%CI 1.64, 9.87), and older age (≥35 years old vs. 18–24 years, aOR = 0.58, 95%CI 0.37, 0.89) (Table 2).

### Prediction of chlamydia re-testing and chlamydia re-infection within 1 year using machine learning approaches

**Figure 1:**
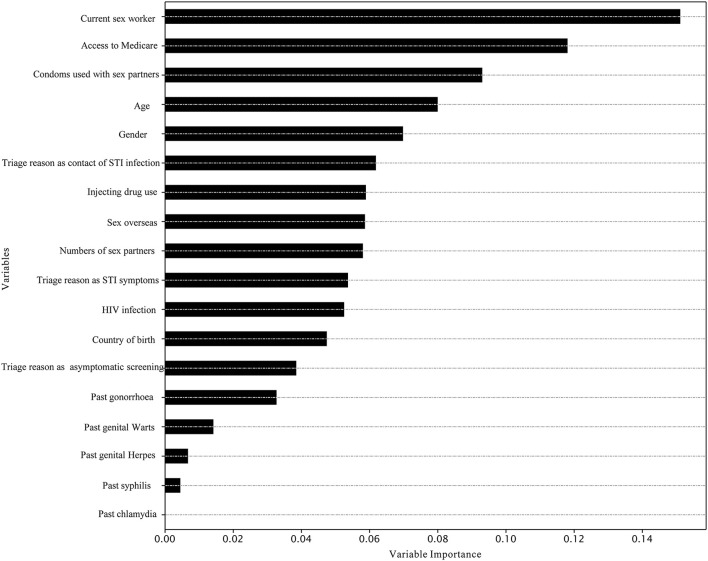
Variable importance in the prediction of chlamydia re-testing within 1 year among heterosexuals by XGBoost.

The best model for predicting chlamydia re-testing within 1 year among heterosexuals with chlamydia was the XGBoost [mean 58.5% (SD 0.1%)]. Adaboost, SVM, LightGBM, and XGBoost performed better than LR in predicting chlamydia re-testing within 1 year among heterosexuals with chlamydia. However, machine learning approaches did not provide a good predictive value for chlamydia re-testing on the testing data (AUC < 60.0%). Further details of model performance are provided in Table 3 and Supplementary Table 1. Variable importance analysis using XGBoost showed that the top 10 identified predictors for chlamydia re-testing within 1 year among heterosexuals with chlamydia included current sex worker, access to Medicare, condoms used with sex partners, age, gender, triage reason as contact of STI infection, injecting drug use, sex overseas, numbers of sex partners, and triage reason as STI symptoms (Figure 1).

**Table 3:** Machine learning model evaluation of chlamydia re-testing and re-infection within1 year among heterosexual individuals with chlamydia on the testing data set (mean/SD).

	**Chlamydia re-testing (*****n*** = **5,806)**		**Chlamydia re-infection (*****n*** = **2,070)**	
	**AUC, %**	**Accuracy, %**	**AUC, %**	**Accuracy, %**
LR	57.6 (1.3)	65.3 (4.1)	57.3 (1.5)	85.2 (1.6)
Adaboost	57.8 (0.4)	65.4 (3.9)	58.3 (1.7)	85.0 (1.7)
SVM	58.1 (2.4)	65.3 (4.4)	47.8 (0.9)	85.2 (1.6)
XGBoost	58.5 (0.1)	64.9 (3.1)	58.5 (1.1)	85.2 (1.6)
RF	57.0 (1.1)	64.1 (2.6)	56.5 (2.0)	85.2 (1.6)
KNN	53.7 (0.1)	63.0 (3.2)	49.5 (2.5)	84.6 (2.3)
Gaussian NB	56.2 (0.6)	61.1 (2.1)	56.7 (1.6)	59.0 (29.7)
GBM	56.9 (1.5)	64.9 (4.4)	56.6 (3.3)	79.2 (9.9)
LightGBM	58.5 (0.2)	63.3 (2.5)	55.3 (1.7)	85.2 (1.6)
MLP	54.7 (3.8)	64.2 (4.7)	55.8 (1.4)	85.3 (1.3)

SD, standard deviation; AUC, area under the receiver operating characteristics curve; LR, Logistic Regression; KNN, K-Nearest Neighbor; AdaBoost, AdaBoost classifier; SVM, SVM with a Radial Basis Function Kernel; GaussianNB, Gaussian Naive Bayes; GBM, Gradient Boosting Machine; LightGBM, Light Gradient Boosting Machine, XGBoost, Extreme Gradient Boosting; RF, Random Forest; MLP, and multi-layer perceptron.

**Figure 2:**
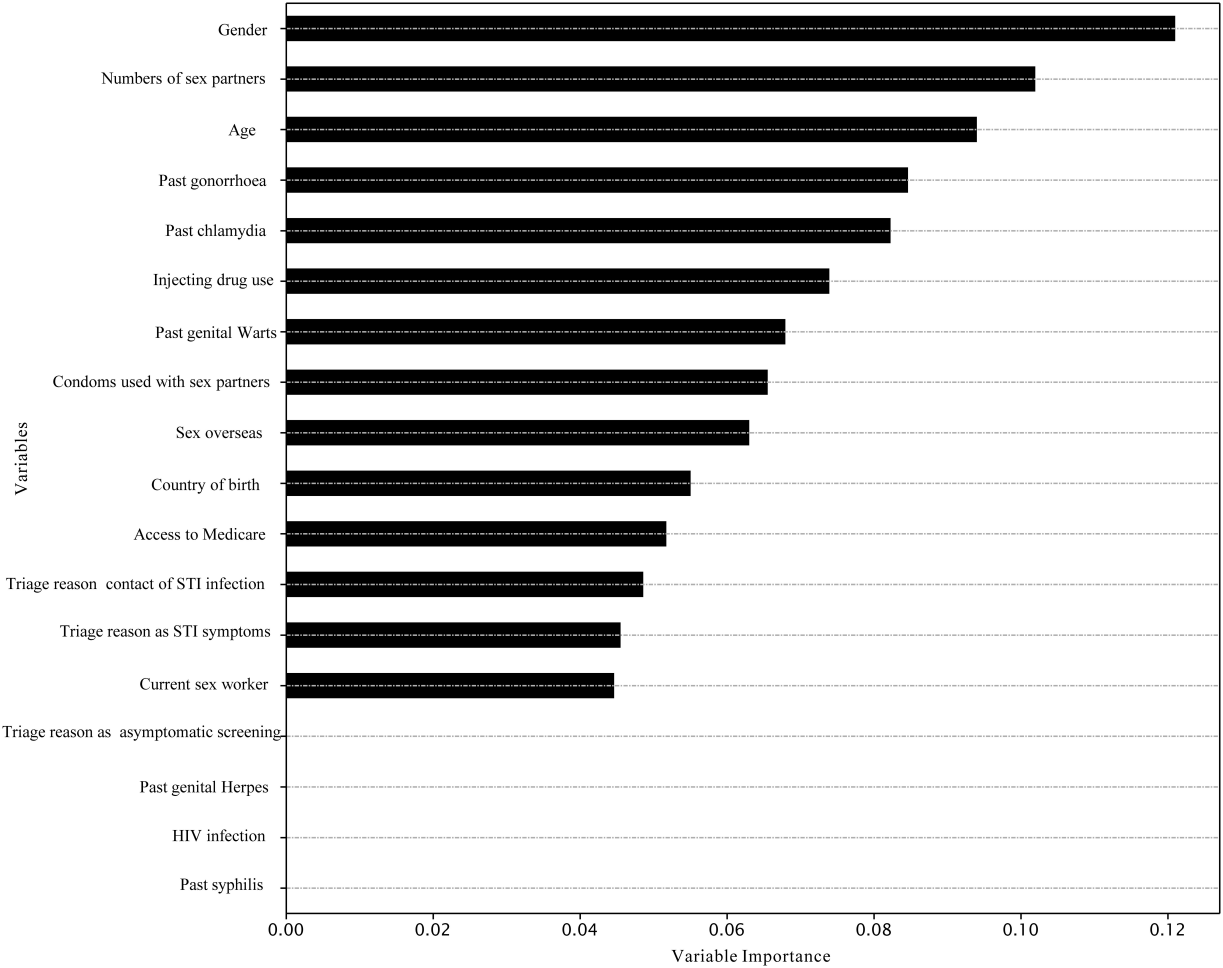
Variable importance in the prediction of chlamydia re-infection within 1 year among heterosexuals by XGBoost.

The best model for predicting chlamydia re-infection within 1 year among heterosexuals with chlamydia was the XGBoost [mean 58.5% (SD 1.1%)]. Adaboost and XGBoost performed better than LR in predicting chlamydia re-infection within 1 year among heterosexuals with chlamydia. However, machine learning approaches did not provide a good predictive value for chlamydia re-infection on the testing data (all AUC < 60.0%). Further details of model performance are provided in Table 3 and Supplementary Table 1. Variable importance analysis using XGBoost showed that the top 10 identified predictors for chlamydia re-infection within 1 year among heterosexuals with chlamydia included gender, number of sex partners, age, past gonorrhea, past chlamydia, injecting drug use, past genital warts, condoms used with sex partners, sex overseas, and country of birth (Figure 2).

## Discussion

To our knowledge, this is the first study using machine learning approaches to predict chlamydia re-testing and re-infection within 1 year among heterosexuals with chlamydia. Our study found a relatively low chlamydia re-testing rate (36%) within 1 year among heterosexuals with chlamydia. Our finding was similar to a previous study reporting that about 40% were re-tested within 12 months in 25 general practice clinics in the Australian Collaboration for Chlamydia Enhanced Sentinel Surveillance system (12). Our study also found a relatively high rate of chlamydia re-infection (15%) within 1 year among heterosexuals with chlamydia. Our result is consistent with previous studies reporting that the chlamydia re-infection rate was about 10–20% of patients within 12 months (4–8). The low rate of chlamydia re-testing and high rate of chlamydia re-infection suggest that further targeted and personalized interventions are warranted among heterosexuals with chlamydia. Our logistic regression analysis showed the determinants of chlamydia re-testing and re-infection within 1 year among heterosexuals with chlamydia. Our machine learning predictive models proposed a new way of using risk prediction models to increase the low chlamydia re-testing rate and reduce the high rate of chlamydia re-infection. Despite the relatively poor prediction of chlamydia re-testing and re-infection among heterosexuals, our findings suggest that researchers could develop a wide range of machine learning algorithms to find the best algorithm for prediction. Our study also provides implications for further data collection in machine learning analysis. In this study, we used routinely collected data from the clinical setting. To improve the performance of learning models in predicting chlamydia re-testing and re-infection, researchers could pay more attention to the quality, comprehensiveness, and accuracy of data collection to avoid “garbage in, garbage out” (29). Our study provides implications for selecting machine learning algorithms and predictors for future machine learning studies in predicting chlamydia re-testing and re-infection among heterosexuals with chlamydia. Better targeting interventions for those at increased risk of re-infection may help reduce their risk or encourage re-testing to diagnose and treat their infection earlier.

We aimed to develop machine learning models to predict chlamydia re-testing behavior within 1 year among heterosexuals with chlamydia using routine questions in the clinical setting. There are already some studies that have utilized machine learning algorithms to predict sexual-health behaviors, including sexual recidivism (30), sexual desire (31), reasons for not using a condom (32), and HIV risk behaviors (33). However, limited studies use machine learning algorithms to predict sexual health service utilization behaviors. We noted a previous study that reported that machine learning approaches could predict HIV/STI re-testing after receiving clinic reminder messages among MSM (24). Another study used machine learning algorithms to predict HIV testing behavior among participants from substance use disorder treatment programs (34). Our study is the first to use machine learning approaches to predict chlamydia re-testing. Therefore, it is difficult to compare our results to others. Despite the poor prediction of chlamydia re-testing behavior, developing good predictive models for this is still necessary and important. Predicting sexual healthcare utilization behaviors (e.g., future chlamydia re-testing) may benefit the management and planning of sexual health services for high-risk populations or focus prevention interventions in advance. We hope our work could encourage more machine learning research to predict chlamydia re-testing behavior among heterosexuals with chlamydia.

We intended to develop predictive models for chlamydia re-infection within 1 year among heterosexuals using machine learning approaches. Predicting future chlamydia re-infection could inform prioritizing interventions. A previous study used machine learning algorithms that could predict chlamydia (AUC = 0.67) acquisition within 1 year among males and females (28). However, our machine learning predictive models indicated that existing self-reported and routinely collected EHRs data could not provide a high predictive value for chlamydia re-infection events within 1 year among heterosexuals with chlamydia (AUC < 0.6). Despite this, our study still provides implications for future machine learning studies focusing on predicting chlamydia re-infection. For example, our results showed that Adaboost, and XGBoost performed better than conventional logistic regression in predicting chlamydia re-infection within 1 year among heterosexuals. Besides, the low predictive performances suggest that existing self-reported and routinely collected EHR data may not include some important predictors for chlamydia re-infection, such as sexual networks and background chlamydia prevalence. Another machine learning study indicated that their models lacked data for specific sexual practices (23). Our models did not include other potential factors because these data were not routinely collected EHRs data in the clinic, such as employment status (8), cervical infection, contact with uncircumcised partners and resuming sex with an untreated partner (35, 36).

Better targeting of interventions to improve re-testing is needed, especially for those with an increased likelihood of chlamydia re-infection. Our study findings are consistent with previous studies, which also found chlamydia re-testing was associated with higher numbers of sex partners (37) and among heterosexual females (38). We also observed that sex workers showed a higher re-testing rate, probably because they had a legal requirement for 3 monthly testing. Our variable importance analyses suggest other possible predictors for future predictive models of chlamydia re-testing using machine learning approaches. The top identified predictors for chlamydia re-testing were being a current sex worker, access to Medicare, condoms used with sex partners, age, gender, triage reason as contact of STI infection, injecting drug use, sex overseas, number of sex partners, and presentation as symptomatic. We hope our preliminary work encourages more machine learning research to explore the effect of introducing additional predictors on predicting chlamydia re-testing among heterosexuals.

Our study found that the chlamydia re-infection rate was greater among individuals of younger age (18–24 years), male gender, and living with HIV. These factors were consistent with previous studies on risk factors for chlamydia infection. A previous study showed that people living with HIV had more repeat chlamydia infections in the Netherlands (39). Young age at first infection was associated with an increased risk of subsequent chlamydia infection within 1 year among female adolescents in the US (26). A previous study found that being a sex worker was associated with chlamydia (40). These findings may provide clinical and public health researchers and policymakers with a deeper understanding of the drivers of chlamydia re-infection among heterosexuals at the population level. Besides, our variable importance analyses suggest possible important predictors for future predictive models of chlamydia re-infection using machine learning approaches. Consistent with previous studies on risk factors of chlamydia infection, we found that factors such as condom use (41), having sex with a female or male partner (42), and sex workers (40) were among the important predictors included in the machine learning models.

Our study has some limitations. First, only about 36% of individuals diagnosed with chlamydia returned for another chlamydia test within 1 year. We did not have information on whether the remaining individuals were tested elsewhere, so this may be an underestimate and may lead to a selection bias in our study. Among those retested, about 15% were re-infected. It is possible that those at higher risk of re-infection may be more likely to be retested. Therefore, the chlamydia re-infection (15%) may be overestimated. Second, our findings were from a single sexual clinic; therefore, studies from different settings (e.g., those attending general practice) would be needed to verify our results. Third, the validity of the predictive factors depends on the accuracy of the self-reported information, subject to participants' recall and non-response bias. There has been substantial work on the CASI system to ensure its validity and accuracy (43). Fourth, we did not include gonorrhea and chlamydia coinfection status in this study. A previous study suggested that gonorrhea and chlamydia coinfection may increase the risk of chlamydia re-infection (36). Last, we included data up to May 2020, which might introduce selection bias due to the COVID-19 lockdown in Melbourne. The first COVID-19 lockdown started on March 30 and ended on May 12, 2020. Thus, our study included a small number of clinic consultations during the COVID-19 lockdown period. A previous study showed that the re-testing patterns and sexual practices might have changed due to COVID-19 (44).

## Conclusions

The chlamydia re-testing rate within 1 year was relatively low among heterosexuals with chlamydia; however, the re-infection rate of chlamydia was relatively high. Our study highlights the need for innovative interventions to increase chlamydia re-testing and reduce re-infection among heterosexuals with chlamydia. Our findings elaborate on the determinants of chlamydia re-testing and re-infection within 1 year among heterosexuals with chlamydia. To our knowledge, it is the first demonstration of machine learning algorithms to identify heterosexuals with chlamydia at high risk of re-infection who could benefit from patient-delivered partner therapy. XGBoost model can improve the prediction of chlamydia re-testing and re-infection compared with traditional logistic regression. Our machine learning predictive models may provide a promising way to develop an innovative method to increase the low chlamydia re-testing rate and reduce the high rate of chlamydia re-infection.

## Data availability statement

The data analyzed in this study is subject to the following licenses/restrictions: The data is not publicly available due to privacy or ethical restrictions but will be made available on reasonable request to the corresponding author, with the permission of the Alfred Hospital Ethics Committee. Restrictions apply to the availability of these data, which were used under ethical approval for this study. Requests to access these datasets should be directed to JO, jason.ong@monash.edu.

## Ethics statement

The studies involving human participants were reviewed and approved by the Alfred Hospital Ethics Committee, Australia (project number: 277/20). As this was a retrospective study involving minimal risk to the privacy of the study subjects, informed consent was waived by the Alfred Hospital Ethics Committee. All identifying details of the study subjects were removed before any computational analysis.

## Author contributions

JO, XX, and LZ conceived and designed the study. XX did data cleaning, established the models and coding, and wrote the first draft and editing. EC, JO, and LZ contributed to data cleaning. EC, CF, MC, IA, JG, JH, NC, LZ, and JO contributed to the interpretation of data and manuscript revision. All authors contributed to the preparation of the manuscript and approved the final manuscript.
