# Supplementary material for: Determinants and prediction of Chlamydia trachomatis re-testing and re-infection within 1 year among heterosexuals with chlamydia attending a sexual health clinic
Source: Front Public Health. 2023 Jan 13;10:1031372. doi: 10.3389/fpubh.2022.1031372 (PMC9880158; doi:10.3389/fpubh.2022.1031372)
Supplement: Supplementary file 1 [file Table_1.DOCX]

Supplementary Material

**Supplementary Table 1.** Machine learning model evaluation of chlamydia retesting and reinfection within one year among heterosexuals within one year on the testing data set (mean/SD)

|  | chlamydia retesting (n=5,806) | | chlamydia reinfection (n=2,070) | |
| --- | --- | --- | --- | --- |
|  | Sensitivity, % | Specificity, % | Sensitivity, % | Specificity, % |
| LR | 11.1(2.0) | 94.2(2.3) | 36.9 (6.0) | 63.9 (2.8) |
| RF | 12.6(3.6) | 94.6(3.2) | 17.4 (3.8) | 83.0 (2.1) |
| KNN | 15.2(6.8) | 90.3(5.0) | 39.1 (7.1) | 63.9 (4.1) |
| Gaussian NB | 37.7(12.1) | 71.6(9.2) | 75.1 (5.7) | 23.9 (3.8) |
| GBM | 14.2(1.4) | 92.9(2.0) | 16.9 (4.0) | 83.5 (2.5) |
| Adaboost | 13.7(3.0) | 93.9(1.9) | 30.0 (5.2) | 71.9 (3.1) |
| SVM | 9.2(2.9) | 96.2(1.6) | 14.2 (5.1) | 86.7 (3.2) |
| XGBoost | 12.1(2.6) | 95.2(2.2) | 15.5(1.3) | 84.0(0.6) |
| MLP | 13.2(3.3) | 93.0(3.7) | 17.7 (5.0) | 83.9 (2.1) |

Note: SD: standard deviation. CI: Confidence Interval. Logistic Regression (LR), K-Nearest Neighbour (KNN), AdaBoost classifier (AdaBoost), SVM with a Radial Basis Function Kernel (SVM), Gaussian Naive Bayes (GaussianNB), Gradient Boosting Machine (GBM), Extreme Gradient Boosting (XGBoost), Random Forest (RF), and multi-layer perceptron (MLP)
